# Alternative implication of CXCR4 in JAK2/STAT3 activation in small cell lung cancer

**DOI:** 10.1038/sj.bjc.6605068

**Published:** 2009-05-19

**Authors:** M Pfeiffer, T N Hartmann, M Leick, J Catusse, A Schmitt-Graeff, M Burger

**Affiliations:** 1 Department of Internal Medicine, Freiburg University Hospital, Freiburg, Germany; 2 Laboratory for Immunological and Molecular Cancer Research, IIIrd Medical Department, University Hospital, Salzburg, Austria; 3 Faculty of Biology, University of Freiburg, Freiburg, Germany; 4 Department of Pathology, Freiburg University Hospital, Freiburg, Germany

**Keywords:** chemokine receptor, signal transduction, CXCR4, STAT3, lung cancer, SCLC

## Abstract

Small cell lung cancer (SCLC) is an aggressive, rapidly metastasising tumour. Previously, we demonstrated the influence of CXCL12–CXCR4 interaction on processes involved in metastasis and chemoresistance in SCLC. We show here that STAT3 is expressed in both primary SCLC tumour tissues and SCLC cell lines. We investigated the function of STAT3 upon CXCL12 stimulation in SCLC cell lines. Small cell lung cancer cell lines present constitutive phosphorylation of STAT3, and in the reference cell lines NCI-H69 and NCI-H82 constitutive phosphorylation was further increased by CXCL12 stimulation. Further investigating this signalling cascade, we showed that it involves interactions between CXCR4 and JAK2 in both cell lines. However CXCL12-induced adhesion to VCAM-1 could be completely inhibited by the JAK2 inhibitor AG490 only in NCI-H82. Furthermore, CXCR4 antagonist but not AG490 inhibited cell adhesion whereas both antagonisms were shown to inhibit growth of the cells in soft agar, indicating the central involvement of this signalling in anchorage-independent growth of SCLC cells. Most interestingly, while using primary tumour material, we observed that in contrast to non-small-cell lung cancer samples from primary tumour tissues, all analysed samples from SCLC were strongly positive for tyrosine-phosphorylated STAT3. Taken together, these data indicate that STAT3 is constitutively phosphorylated in SCLC and is important in SCLC growth and spreading thus presenting an interesting target for therapy.

Small cell lung cancer (SCLC) is a highly fatal cancer due to early and widespread metastasis and development of resistance to chemotherapy (Hoffman *et al*, 2000). Despite initial sensitivity to chemotherapy, SCLC almost invariably relapses, and the patients' 5-year survival rate is less than 5% (Chute, 2006). According to the cancer homing theory, malignant cells of different solid tumours (e.g. breast and prostate cancer) might use trafficking mechanisms of haematopoietic stem cells to reach their distinct target tissues (Wang *et al*, 2006). The chemokine receptor CXCR4 (CD184) and its ligand CXCL12 (SDF-1*α*) are essential for the homing and bone marrow interaction of haematopoietic stem cells (Peled *et al*, 1999, Peled *et al*, 2000; Ratajczak *et al*, 2006). Indeed, SCLC cell lines and primary tumour cells express high levels of CXCR4 (Kijima *et al*, 2002; Burger *et al*, 2003), and CXCR4 ligand CXCL12 is constitutively expressed by bone marrow stromal cells (Muller *et al*, 2001). Previously, we have shown that CXCR4 mediates important functions for SCLC metastasis to stromal microenvironments, for example adhesion of SCLC cells to immobilised extracellular matrix proteins and stromal cells by activation of *β*1-integrins, leading to cell adhesion-mediated drug resistance that protects SCLC cells from chemotherapeutic treatment (Sethi *et al*, 1999; Burger *et al*, 2003; Hartmann *et al*, 2005).

Stimulation with CXCL12 leads to activation of various pathways in SCLC cells, and CXCR4 has also been reported to activate the JAK/STAT3 pathway (Vila-Coro *et al*, 1999; Ahr *et al*, 2005). Signal transducers and activators of transcription (STAT) proteins are latent transcription factors that become activated by phosphorylation on a specific tyrosine residue by JAK-family kinases (Darnell, 1997). Upon cytokine-induced receptor activation, JAKs phosphorylate monomeric STAT proteins, which dimerise and translocate to the nucleus in a highly regulated way leading to gene transcription (Reich and Liu, 2006). In many cancers, STAT3 is constitutively activated, and aberrant STAT3 signalling is implicated as an important process in malignant transformation (Bromberg *et al*, 1999) and induction of angiogenesis (Niu *et al*, 2002). The tight association of STAT3 activation with transformation and tumour progression makes it an attractive molecular target for novel cancer therapeutics development and many substances have been tested for their effect on STAT3 (Deng *et al*, 2007). Although a link was found between CXCR4 and STAT3 in different cells, such as haematopoietic progenitor cells (Zhang *et al*, 2001) or a fibrosarcoma cell line (Soriano *et al*, 2002), there are no reports on STAT3 signalling in SCLC so far. Because of previously reported links between CXCR4 and JAK/STAT signalling and potential of this pathway to provide drug targets, we were interested in whether this pathway is involved in SCLC.

In this study we demonstrate that, as shown for many tumours, STAT3 is strongly phosphorylated in tumour tissue samples from patients with SCLC in comparison to samples from patients with NSCLC (non-small-cell lung cancer). We report possible increased CXCL12-induced STAT3 phosphorylation as well as lack of CXCL12 effect on STAT3 phosphorylation depending on the cell line. Further investigating this mechanism, we showed that JAK2/STAT3 activation can be involved in anchorage-dependent cell growth but is not involved in the adhesion of SCLC cells to stromal cells.

## Materials and methods

### Cell culture, chemokines, antibodies, and inhibitors

Small cell lung cancer cell lines (NCI-H69, NCI-H82, NCI-H146, NCI-H345, NCI-H446, NCI-H510A, and NCI-N592) and the murine stromal cell line M2-10B4 were obtained from the American Type Culture Collection (Manassas, VA, USA). The cells were maintained in RPMI-1640 medium containing 10% FCS, 1% penicillin, and streptomycin (Gibco-BRL, Grand Island, NY, USA). Cell viability was determined with Trypan blue staining. Synthetic human CXCL12 and JAK2 antibodies were purchased from Upstate Biotechnology (Lake Placid, NY, USA). Antibodies against STAT3 and phospho-STAT3 (Y705) were purchased from Cell Signalling Technology Inc. (Beverly, MA, USA). Antibody against CXCR4 was purchased from R&D Systems (Wiesbaden, Germany). Antibody against CD56 was obtained from AbD Serotec (Duesseldorf, Germany). Antibodies against integrin subunits were purchased from Chemicon (Schwalbach, Germany). The inhibitor TN14003 was developed and kindly provided by N Fujii (Kyoto, Japan; original publication, MRI, *Molecular Cancer Therapeutics*, 2003). Pertussis toxin was obtained from Merck Biosciences (Darmstadt, Germany). AG490 and anti-integrin antibodies were purchased from Calbiochem (Bad Soden, Germany).

### Nuclear extracts and western blotting

Small cell lung cancer cells were serum starved for 3 h before stimulation with CXCL12. Cells were preincubated with AG490 for 60 min and stimulated with CXCL12. Cells (5 × 10^6^) per sample were lysed under hypotonic conditions to spare the nuclear membrane (10 mM HEPES (pH 8.0), 5 mM KCl, 0.1 mM EDTA, 0.1 mM EGTA, 1 mM Na_3_VO_4_, 1 mM DTT, 0.5 mM PMSF, protease inhibitor mix (‘complete’; Roche Applied Science, Penzberg, Germany). The nuclear fraction was separated by centrifugation and was lysed subsequently (20 mM HEPES (pH 8.0), 400 mM NaCl, 1 mM EDTA, 1 mM EGTA, 2 mM Na_3_VO_4_, 1 mM DTT, 0.5 mM PMSF, protease inhibitor mix).

Equal amounts of nuclear fractions were separated by 10% SDS–polyacrylamide gel electrophoresis (PAGE) and transferred onto nitrocellulose membranes. Immunostaining was performed using antibodies against STAT3 or phospho-STAT3 (Y705). Bands were visualised using horseradish peroxidase-conjugated secondary antibody and the enhanced chemiluminescence system (Amersham Biosciences, Freiburg, Germany).

### Co-immunoprecipitation

Cells (5 × 10^6^) per sample were serum starved, stimulated with CXCL12, centrifuged, and resuspended in 400 *μ*l lysis buffer (20 mM Tris-HCl (pH 8.0), 150 mM KCl, 1 mM EDTA, 1 mM Na_3_VO_4_, 1 mM PMSF, 1% Triton X-100, protease inhibitor mix). CXCR4 antibody was added overnight. Immunoprecipitates were collected with 30 *μ*l Protein G sepharose beads (Amersham Biosciences). They were washed three times with lysis buffer and once with PBS, fractionated with SDS–PAGE and immunoblotted with a JAK2 antibody.

### SCLC adhesion to immobilised collagen, fibronectin, and VCAM-1

Adhesion assays to proteins of the extracellular matrix were performed as described before (Hartmann *et al*, 2005). Briefly, 96-well plates were coated with collagen type I (5 *μ*g ml^−1^), fibronectin (10 *μ*g ml^−1^), and VCAM-1 (1.3 *μ*g ml^−1^) overnight. After washing steps, CXCL12 was co-immoblised for 30 min at room temperature (8 *μ*g ml^−1^) and the wells were blocked for 60 min with 1% tissue culture BSA (Sigma, Munich, Germany) in PBS. Small cell lung cancer cells were pretreated with AG490 (100 *μ*M) or DMSO (0.2%) for 60 min and added to the wells in quadruplicates and allowed to adhere for 30 min at 37°C in adhesion buffer (HBSS buffered with HEPES and supplemented with 0.5% BSA). The number of adherent cells was determined using the CyQUANT Cell Proliferation Assay (Molecular Probes, Eugene, OR, USA) and fluorescence was measured by a microtiter plate fluorometer (Spectra Max Gemini XS; Molecular Devices, Munich, Germany). Five independent experiments were performed.

### SCLC cell adhesion to stromal cells

The murine stromal cell line M2-10B4 was seeded the day before the assay onto 24-well plates at a concentration of 1.5 × 10^5^ cells per well in RPMI-1640 supplemented with 10% FCS. Small cell lung cancer cells were preincubated with TN14003 (100 *μ*g ml^−1^; 30 min), AG490 (100 *μ*M; 60 min), DMSO (0.2%; 60 min), l cell layer at a concentration of 1 × 10^6^ cells per well. After 3 h at 37°C in 5% CO_2_, the wells were washed vigorously three times from four directions with 1 ml RPMI to remove non-adhering cells. The remaining cells, including stromal cells and adhering SCLC cells, were detached in trypsin/EDTA followed by three washing steps in FACS medium (RPMI+0.5% BSA). To distinguish SCLC cells from stromal cells, we labelled them with an anti-CD56-PE antibody for 30 min at 4°C and measured them by flow cytometry (FACSCalibur; Becton Dickinson, Heidelberg, Germany). Thus, increased proportion of CD56+ cells in the total cell suspension results from pre-trypsin treatment adherent cells. Samples were analysed in duplicates and each experiment was independently performed five times.

### Expression of integrin subunits and VCAM-1

Expression of integrin subunits was determined using monoclonal CD29, CD49a, CD49b, CD49c, CD49d, and CD49e antibodies and the appropriate isotype controls (Becton Dickinson). Small cell lung cancer cells were adjusted to a concentration of 10^6^ cells per ml in PBS+0.5% BSA and stained with saturated antibody concentrations for 20 min at 4°C. After washing the cells twice, integrin expression was analysed by flow cytometry.

Expression of soluble VCAM-1 in cell culture supernatants was detected by an ELISA provided by R&D Systems (Quantikine Mouse sVCAM-1 Immunoassay), according to the manufacturer's instructions.

### Soft agar assay

Small cell lung cancer cells (1 × 10^4^) were singularised in 2 ml RPMI-1640 containing 10% FCS and 0.3% agarose. Before pouring the cells onto a bottom layer of 0.6% soft agar in 12-well plates, TN14003 (100 *μ*g ml^−1^) or AG490 (100 *μ*M) were added. Untreated cells and DMSO-treated cells (0.2%) were used as controls. The cells were fed three times a week with fresh medium, containing the appropriate inhibitors. Cell colonies were photographed after 2 weeks.

### Immunohistochemistry

Tissue samples of primary tumours from patients suffering from SCLC (*n*=10) and NSCLC (*n*=13) were fixed in formalin and embedded in paraffin. Sections (3 *μ*m) were dried, dewaxed in xylene, and rehydrated through graded alcohol to water. Antigen retrieval was carried out by boiling the samples at pH 6.1. Antigens were stained using the alkaline phosphatase anti-alkaline phosphatase method (Dako, Hamburg, Germany) as described by the manufacturer. Antibodies against STAT3 or phospho-STAT3 (Y705) were used at a concentration of 1 : 100.

### Statistical analysis

For statistical analysis, the paired Student's *t*-test for comparison of two groups was used. Analyses were performed with the statistic tool of Origin 7G (OriginLab Corporation, Northampton, MA, USA).

## Results

### Constitutive and inducible STAT3 in SCLC cell lines

**Figure 1:**
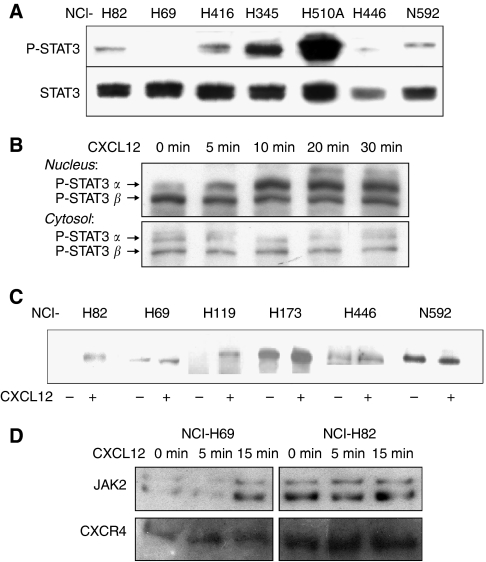
Characterisation of JAK2/STAT3 pathway in SCLC cells. Nuclear extracts of untreated SCLC cells were analysed by western blot analysis and showed STAT3 phosphorylation at different degrees. (**A**) CXCL12-induced phosphorylation of STAT3 occurred in a time-dependent manner, as observed in western blot analysis with nuclear extracts of NCI-H69 treated with 100 ng ml^−1^ CXCL12 (**B**). All tested cell lines but NCI-N592 showed induction of STAT3 phosphorylation by CXCL12 stimulation (**C**). JAK2 was stained in a western blot analysis following co-immunoprecipitation with a CXCR4 antibody after stimulating NCI-H69 (left panel) and NCI-H82 (right panel) with 100 ng ml^−1^ CXCL12 for the indicated period of time (**D**). Each blot shown is representative for at least three independent experiments.

To assess the levels of phosphorylated STAT3 in SCLC, we prepared and examined nuclear extracts from seven unstimulated SCLC cell lines by western blot analysis using an anti-phospho-STAT3 antibody. As a control, total nuclear STAT3 was stained. We found constitutive STAT3 phosphorylation in most of the investigated cell lines, but at variable levels. The cell line NCI-H510A displayed exceptionally strong constitutive STAT3 phosphorylation whereas NCI-H69 displayed the lowest level with slight to undetectable constitutive expression (Figure 1A). As NCI-H69 and NCI-H82 are the most commonly investigated SCLC cell lines and displayed rather weak constitutive activation, we further used these two cell lines to determine whether CXCL12 is able to directly activate JAK2/STAT3 signalling pathways in SCLC cells. To do so we measured levels of tyrosine-phosphorylated STAT3 in nuclear extracts after CXCL12 treatment. STAT3 exists in different isoforms; STAT3*α* is the most commonly described so far. STAT3*α* was phosphorylated upon CXCL12 stimulation in a time-dependent manner with a maximum after 10 min, still showing a strong signal after 30 min, reduced cytosolic effect might be the result of the low representation of STAT3 in this pool (Figure 1B). CXCL12-induced tyrosine phosphorylation of STAT3 could be observed in SCLC cells lines NCI-H69, NCI-H82, NCI-H119, NCI-H173, and NCI-H446, but not in NCI-N592 (Figure 1C). High expression of CXCR4 has however been described in NCI-N592 (Burger *et al*, 2003) underlying the heterogeneity of SCLC cell lines and reflecting heterogeneity among SCLC diagnosed tumours themselves.

To directly analyse the association of JAK2 with CXCR4 upon CXCL12 stimulation, we performed co-immunoprecipitation assays with NCI-H69 and NCI-H82. In both cell lines, constitutive association of JAK2 with CXCR4 was observed, however with a lower intensity for NCI-H69. Association of JAK2 with CXCR4 increased upon stimulation with CXCL12 with a maximal association observed after 15 min (Figure 1D). In contrast, NCI-H82 showed a strong constitutive association of JAK2 to CXCR4 that could not be further increased by CXCL12 stimulation. The observation of two bands of phosphorylated JAK-2 was unexpected and is likely to result from protein degradation.

### Expression and functions of *β*1-integrins in SCLC cell lines NCI-H69 and NCI-H82

**Figure 2:**
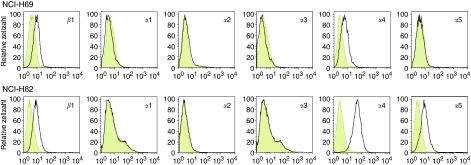
Expression of integrin subunits in SCLC cell lines. Fluorescence histograms depicting the expression of *β*1-integrin (CD29) and *α*1-, *α*2-, *α*3-, *α*4-, *α*5-integrin (CD49a, CD49b, CD49c, CD49d, CD49e) subunits by SCLC cell lines NCI-H69 and NCI-H82 are shown by black lines. The respective isotype controls are represented by grey shaded histograms.

We investigated further the differences between NCI-H69 and NCI-H82 activation focusing on another family of proteins: integrins. Integrins form a family of heterodimeric proteins (*α*- and *β-*subunits). They are cell adhesion molecules that mediate cell–extracellular matrix as well as cell–cell interactions. It has been demonstrated that *β*1-integrin signalling leading to malignant cell adhesion is involved in resistance of SCLC cells against chemotherapy (Sethi *et al*, 1999). Both SCLC cell lines analysed, NCI-H69 and NCI-H82, were positive for *β*1-integrin and negative for *α*1-, *α*2-, and *α*3-integrins, but differed in expression of *α*4- and *α*5-subunits. In contrast to NCI-H69 that did not express any *α*5, NCI-H82 was positive for this subunit. Integrin *α*4 was expressed in NCI-H82 more abundantly than in NCI-H69 (Figure 2). Interestingly, *α*4-*β*1 integrin, also called VLA4 (alpha4-beta1 integrin), is known to be involved in cell-adhesion mediated drug resistance of tumours (Sanz-Rodríguez *et al*, 2001).

### Adhesion of NCI-H82 to VCAM-1 is dependent on JAK2

Previously, we have shown that immobilised CXCL12 induces VLA-4-mediated adhesion of SCLC cell lines to immobilised VCAM-1 and fibronectin, which could be inhibited by pertussis toxin and rho-kinase inhibitors (Burger *et al*, 2003; Hartmann *et al*, 2005). Here we investigated the influence of the JAK2/STAT3 pathway on this process. Proteins of the extracellular matrix and CXCL12 were co-immobilised on 96-well plates and the adhesion rate of SCLC cells was determined.

**Figure 3:**
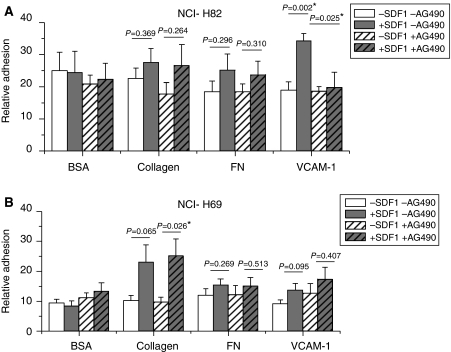
Adhesion to extracellular matrix proteins. Relative adhesion rates of NCI-H82 (**A**) and NCI-H69 (**B**) to VCAM-1, fibronectin and collagen I measured after 30 min are shown for untreated controls, cells stimulated with co-immobilised CXCL12 (8 *μ*g ml^−1^), cells preincubated with AG490 (60 min, 100 *μ*M), and cells that were both preincubated with the inhibitor and stimulated by CXCL12. Error bars represent s.e.m. Differences were accepted as significant when *P*<0.05 (^*^), *n*=5, each experiment in quadruplicates.

The cell line NCI-H82 showed strongest CXCL12-mediated adhesion to VCAM-1, which could be fully reversed by preincubation with JAK2 inhibitor AG490 (Figure 3A). Adhesion of this cell line to collagen and fibronectin was weaker and there was no influence by AG490. Significant increases by CXCL12 stimulation were reached only in presence of VCAM-1 (*P*=0.002), this induction was significantly decreased after treatment with AG490. In contrast, CXCL12 induced NCI-H69 cell adhesion to collagen (*P*=0.065) whereas CXCL12-mediated adhesion to VCAM-1 and fibronectin was lower (*P*=0.095) and significantly reduced compared to results obtained for NCI-H82 cells (*P*=0.002). This correlates to the VLA-4 expression levels of the different cell lines, and the low adhesion of NCI-H69 can be explained by the low VLA-4 expression of this cell line. Basal level of adhesion is markedly different in both cell lines. We could not detect any inhibitory influence of AG490 on CXCL12-triggered adhesion of NCI-H69 cells (Figure 3B). To compare directly the treatment effects in both cell lines, we expressed the results in function of the basal levels of adhesion obtained with control BSA treatment. Results are shown in the Table 1 and illustrate observations described previously, further showing the differences after treatment by AG490 in each cell lines (shaded results).

### Adhesion of SCLC cell lines to the stromal cell line M2-10B4

**Figure 4:**
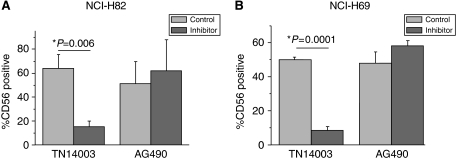
Adhesion to stromal cell line M2-10B4. NCI-H82 (**A**) and NCI-H69 (**B**) were allowed to adhere to M2-10B4 cells for 3 h after pretreatment with AG490 (100 *μ*M, 60 min) or TN14003 (100 *μ*g ml^−1^, 30 min), respectively, then submitted to vigorous washes to remove non-adherent cells from the medium, the whole-cell complex was then submitted to trypsinisation to put it in suspension before assessment of the proportion of each cell type by FACS (*n*=3).

Previously we demonstrated that VLA-4 blocking by anti-*α*4 antibody and CXCR4 blocking by the antagonist TN14003 inhibited SCLC cell adhesion to stromal cells (Hartmann *et al*, 2005). TN14003 is derived from T140, a horseshoe crab self-defence peptide-derived CXCR4 antagonist; TN14003 is serum stable and more specific variant of T140 (Tamamura *et al*, 2004). In addition to CXCL12, soluble VCAM-1 is secreted by M2-10B4: using ELISA we determined that at least 2.38±0.66 ng ml^−1^ VCAM-1 was present in our supernatants (four independent assays in duplicates) as already observed (Hartmann *et al*, 2005). Due to the observed AG490-mediated inhibition of NCI-H82 adhesion to VCAM-1 and the detected VCAM-1 secretion of stromal cells, we investigated if AG490 treatment inhibits NCI-H69 and NCI-H82 adhesion to stromal cells. However, in the more complex environment of stromal interaction, JAK2 blockage alone was not able to inhibit the SCLC cell adhesion to the stroma, whereas CXCR4 inhibition by TN14003 was fully efficient (Figure 4), demonstrating activation of branched pathways triggered by CXCL12 binding to CXCR4.

### Importance of the CXCR4 and JAK2/STAT3 pathway in anchorage-independent growth

**Figure 5:**
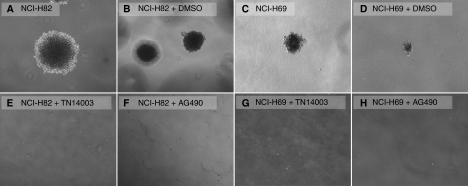
Anchorage-independent growth in soft-agar. SCLC cells NCI-H82 (**A**, **B** and **E**, **F**) and NCI-H69 (**C**, **D** and **G**, **H**) were singularised, put in a 0.3% agarose gel and observed in the absence (**A**, **C**) or presence of TN14003 (100 *μ*gml^−1^) (**E**, **G**) and AG490 (100 *μ*M) (**F**, **H**). Appropriate controls were done with DMSO (**B**, **D**) to rule out any involvement of this solvant in the observed results. Characteristic cell aggregates were photographed after 14 days (*n*=3).

The anchorage-independent growth of SCLC cells was determined in a soft agar assay (Missale *et al*, 1998). In absence of inhibitors and in the DMSO control, the cells were able to build large aggregates in the soft agar during the 2-week observation period. In contrast, there was no proliferation in the presence of the CXCR4 inhibitor TN14003, which suggests a contribution for this chemokine receptor in this process. There was also a complete inhibition by the JAK2 inhibitor AG490, suggesting importance of this pathway in anchorage-independent growth (Figure 5). In the first week, both the inhibited cells and controls looked quite similar under the microscope except for the fact that the inhibited ones did not proliferate. However, after 7–10 days cells inhibited with TN14003 and AG490 began to change their morphology. They became rounder, sharper circumscribed, and finally darker in appearance than the controls.

### STAT3 is phosphorylated in primary tumours from patients with SCLC

**Figure 6:**
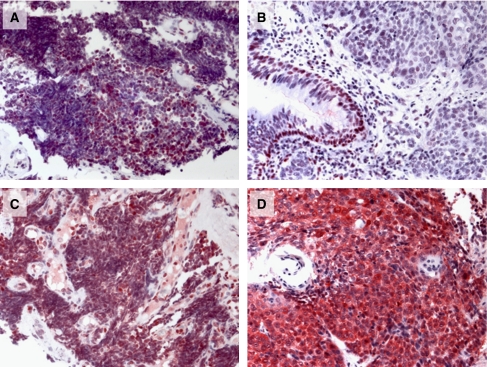
Representative immunohistochemical staining of primary tumour samples from 10 patients with SCLC (**A** and **C**) and 13 patients with NSCLC (**B** and **D**). Slides were stained with phospho-Tyr705-STAT3 antibody (upper row: **A** and **B**) and STAT3 antibody (inferior row: **C** and **D**), respectively.

To assess the relevance of these observations in a pathophysiological context, we investigated STAT3 phosphorylation in SCLC and compared it to NSCLC. Tissue samples of primary tumours from patients suffering from SCLC (*n*=10) and NSCLC (*n*=13) were stained with anti-phospho-Tyr705 STAT3 and anti-STAT3 antibodies in immunohistochemistry. Samples from patients suffering from SCLC (10 out of 10) were found to be strongly positive for phosphorylated STAT3, whereas samples from patients suffering from NSCLC (13 out of 13) showed significantly phosphorylated STAT3 only in basal layers of the respiratory epithelia and alveolar macrophages, but not within the tumour tissue. For the detection of unphosphorylated STAT3, samples from both SCLC and NSCLC were stained equally using an anti-STAT3 antibody (Figure 6).

## Discussion

The JAK2/STAT3 pathway is involved in various signalling cascades, including signalling of receptor tyrosine kinases and G-protein-coupled receptors. Activation of the JAK/STAT pathway is linked to proliferation, and constitutive activation of both JAK2 and STAT3 is described to be important in malignant transformation and tumourigenesis (Bromberg *et al*, 1999; Levy and Darnell, 2002). STAT3 is focusing more and more interest in cancer therapy as it is often overactivated in cancer. The constitutively active JAK2 mutant V617F is found in many patients with myeloproliferative diseases (Levine and Gilliland, 2007), and constitutively phosphorylated STAT3 is found in many solid tumours and haematopoietic malignancies (Lim and Cao, 2006). The chemokine receptor CXCR4 was described to activate the JAK2/STAT3 pathway in different cell lines (Vila-Coro *et al*, 1999; Soriano *et al*, 2003; Opdam *et al*, 2004; Moriguchi *et al*, 2005) and the intracellular domains responsible for JAK2 binding have been discovered by mutational analysis (Ahr *et al*, 2005). Until now, there has been no report on the activation of the JAK/STAT pathway in SCLC.

In this study, we demonstrate that STAT3 is strongly phosphorylated in primary tumour tissues from patients with SCLC indicating that this pathway might be relevant in pathophysiological processes involved in SCLC. In contrast to this, the primary tumour tissue samples from patients with NSCLC did not show any strong STAT3 activation within the tumour tissue. In NSCLC samples, only alveolar macrophages and basal layers of respiratory epithelia showed strongly phosphorylated STAT3, cell types already known to show phosphorylation of STAT3 due to their proliferative capacity. Reports on constitutive phosphorylation of STAT3 in some NSCLC cell lines (Song *et al*, 2003) and lung adenocarcinomas (Gao *et al*, 2007) are not in contrast with our observation because they reported relatively low percentages of strong phosphorylation and the STAT3 phosphorylation was mainly related to mutations in the EGFR kinase domain and IL-6 production.

Small cell lung cancer tumour and cellular models for *in vitro* investigation have been shown to be heterogeneous (Carney *et al*, 1985); however, some common features can be described: all analysed SCLC cell lines showed constitutive STAT3 phosphorylation, however to various degrees. In addition to the constitutive activation of STAT3, we were able to demonstrate that stimulation of SCLC cell lines with CXCL12 increased the STAT3 phosphorylation in all the cell lines but one. Interestingly, we found only the *α*-isoform of STAT3 to be phosphorylated upon stimulation. Both isoforms are thought to have different functions and to induce transcription of different genes (Chakraborty *et al*, 1996; Maritano *et al*, 2004), but functional differences are poorly understood so far. Nevertheless, exclusive STAT3*α* phosphorylation after IL-6 or IL-10 stimulation has been shown in different cellular models (Maritano *et al*, 2004). Previous studies that describe a CXCR4-mediated STAT3 activation do not differentiate between the isoforms so our results are a new hint that cytokine activation targets *α*-isoform phosphorylation. To focus on this point, we used two SCLC cell lines that showed only low constitutive STAT3 phosphorylation but a clearly inducible phosphorylation in response to CXCL12: NCI-H82 and NCI-H69. The association of JAK2 to CXCR4 was induced by CXCL12 in NCI-H69. In NCI-H82 cell line, the constitutive association of JAK2 and CXCR4 is stronger but cannot be further increased. This seems to go along with a stronger activation of NCI-H82 in functional assays. For example, NCI-H82 showed a higher basal adhesion level in comparison to NCI-H69, and NCI-H82 built larger aggregates in soft agar assays.

CXCR7 is a recently described second receptor for CXCL12 and was shown to promote lung cancer growth *in vivo* (Miao *et al*, 2007) but this receptor might rather modulate CXCR4-mediated responses than act as an independent CXCL12 receptor (Hartmann *et al*, 2008). To assess a potential involvement of CXCR7 in the results observed here, we investigated CXCR7 expression at the surface of NCI-H69, NCI-H82, and NCI-N592, with a particular interest for NCI-N592, where the lack of STAT3 activation upon CXCL12 stimulation could be explained by a CXCR7 scavenging of CXCL12. Expression was shown to be absent or very low in NCI-H82 and NCI-N592 but high in NCI-H69 (data not shown); however, consistent inhibition of CXCL12 activation in NCI-H69 by TN14003 seems to rule out any major involvement of CXCR7 in this mechanism of activation.

To further evaluate the functional relevance of the biochemical proof of CXCL12-induced activation of JAK2 and STAT3 and a constitutive STAT3 phosphorylation *in vivo*, we analysed the effect of the JAK2 inhibitor AG490 on adhesion mediated by CXCR4- and anchorage-independent cell growth. Only a few studies demonstrate an influence of JAK2 in cytokine-stimulated adhesion. One study demonstrated almost complete abrogation of CXCL12-induced adhesion of T cells to VCAM-1 by AG490 (García-Bernal *et al*, 2005). In accordance with these data on haematopoietic cells, we observed a complete inhibition of the CXCL12-induced adhesion of the SCLC cell line NCI-H82 to VCAM-1 by AG490. In contrast, CXCL12-induced VCAM-1 adhesion of NCI-H69 was much lower, due to lower *α*4-integrin expression, and we could not demonstrate an AG490 influence. In addition, although the bone marrow stromal cell line M2-10B4 expresses high amounts of CXCL12 and VCAM-1, we could not abrogate SCLC adhesion to M2-10B4 by AG490. This suggests compensating mechanisms or other more dominant pathways in this complex adhesion model. Besides adhesion, anchorage-independent growth is a major function that promotes tumour growth and metastasis. Anchorage-independent growth was reported to be dependent on CXCR4 by many groups and for numerous malignancies (Ratajczak *et al*, 2006; Wang *et al*, 2006). We analysed anchorage-independent growth in a soft agar assay (Missale *et al*, 1998), where cells proliferate without cell or surface contacts and must overcome contact inhibition. Consistently with reports on other malignancies (Sehgal *et al*, 1998; Huang *et al*, 2005), we demonstrate a complete inhibition of soft agar proliferation in the cell lines NCI-H69 and NCI-H82 by blocking JAK2 or CXCR4 with the inhibitors AG490 and TN14003, respectively.

Taken together, we show high constitutive STAT3 phosphorylation in primary tumour samples from patients with SCLC but not from NSCLC, and constitutive and CXCL12-dependent activation of the JAK2/STAT3 pathway in SCLC cell lines. Furthermore, we have shown here, that although STAT3 is commonly implicated in SCLC biology, at least two different upstream pathways dependent or independent of CXCR4 can be involved in its phosphorylation. This is of particular interest in drug development strategies as both CXCR4 and STAT3 antagonisms have been proposed as broad spectrum target in cancer therapy (Wang *et al*, 2006; Germain and Frank, 2007; Ruffini *et al*, 2007). We show here that SCLC drug development should take into account CXCR4/STAT3 linkage to target more specifically the malignant cells; although JAK2/STAT3 seems to have a dominant function in anchorage-independent cell proliferation, it has a minor function in adhesive processes of SCLC cells where CXCR4 antagonists should prove more efficient. Besides CXCR4 antagonists that are currently investigated in an *in vivo* model for SCLC, STAT3 inhibitors on its own or in combination with CXCR4 inhibitors will be very interesting to consider as therapeutic option for the treatment of patients with SCLC.

## Figures and Tables

**Table 1:**
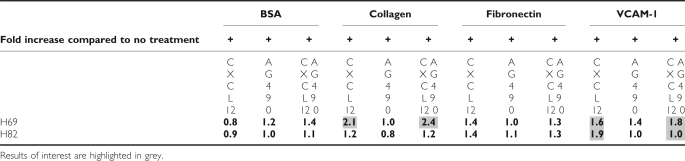
Data are extracted from Figure 3A and B, and represent normalization in reference to basal adhesion in presence of control BSA treatment

## References

[bib1] Ahr B, Denizot M, Robert-Hebmann V, Brelot A, Biard-Piechaczyk M (2005) Identification of the cytoplasmic domains of CXCR4 involved in Jak2 and STAT3 phosphorylation. J Biol Chem 280: 6692–6700 15615703 10.1074/jbc.M408481200

[bib2] Bromberg JF, Wrzeszczynska MH, Devgan G, Zhao Y, Pestell RG, Albanese C, Darnell Jr JE (1999) Stat3 as an oncogene. Cell 98: 295–303 10458605 10.1016/s0092-8674(00)81959-5

[bib3] Burger M, Glodek A, Schmitt-Gräff A, Silberstein LE, Tsukada N, Kipps TJ, Burger JA (2003) CXCR4 (CD184) target small cell lung cancer (SCLC) metastasis to the bone marrow by mediating migration, integrin activation and adhesion to stromal cells. Oncogene 22: 8093–8101 14603250 10.1038/sj.onc.1207097

[bib4] Carney DN, Gazdar AF, Nau M, Minna JD (1985) Biological heterogeneity of small cell lung cancer. Semin Oncol 12: 289–303 2996148

[bib5] Chakraborty A, White SM, Schaefer TS, Ball ED, Dyer KF, Tweardy DJ (1996) Granulocyte colony-stimulating factor activation of Stat3 alpha and Stat3 beta in immature normal and leukemic human myeloid cells. Blood 88: 2442–2449 8839834

[bib6] Chute JP (2006) Stem cell homing. Curr Opin Hematol 13: 399–406 17053451 10.1097/01.moh.0000245698.62511.3d

[bib7] Darnell Jr JE (1997) STATs and gene regulation. Science 277: 1630–1635 9287210 10.1126/science.277.5332.1630

[bib8] Deng J, Grande F, Neamati N (2007) Small molecule inhibitors of Stat3 signaling pathway. Curr Cancer Drug Targets 7: 91–107 17305481 10.2174/156800907780006922

[bib9] Gao SP, Mark KG, Leslie K, Pao W, Motoi N, Gerald W, Travis W, Bornmann W, Veach D, Clarkson B, Bromberg J (2007) Mutations in the EGFR kinase domain mediate STAT3 activation via IL-6 production in human lung adenocarcinomas. J Clin Invest 117: 3846–3856 18060032 10.1172/JCI31871 PMC2096430

[bib10] García-Bernal D, Wright N, Sotillo-Mallo E, Nombela-Arrieta C, Stein J, Bustelo X, Teixidó J (2005) Vav1 and Rac control chemokine-promoted T lymphocyte adhesion mediated by the integrin alpha4beta1. Mol Biol Cell 16: 3223–3235 15872091 10.1091/mbc.E04-12-1049 PMC1165406

[bib11] Germain D, Frank DA (2007) Targeting the cytoplasmic and nuclear functions of signal transducers and activators of transcription 3 for cancer therapy. Clin Can Res 13: 5665–5669 10.1158/1078-0432.CCR-06-2491 17908954

[bib12] Hartmann TN, Burger JA, Glodek A, Fujii N, Burger M (2005) CXCR4 chemokine receptor and integrin signaling co-operate in mediating adhesion and chemoresistance in small cell lung cancer (SCLC) cells. Oncogene 24: 4462–4471 15806155 10.1038/sj.onc.1208621

[bib13] Hartmann TN, Grabovsky V, Pasvolsky R, Shulman Z, Buss EC, Spiegel A, Nagler A, Lapidot T, Thelen M, Alon R (2008) A crosstalk between intracellular CXCR7 and CXCR4 involved in rapid CXCL12-triggered integrin activation but not in chemokine-triggered motility of human T lymphocytes and CD34+ cells. J Leukoc Biol 84: 1130–1140 18653785 10.1189/jlb.0208088

[bib14] Hoffman PC, Mauer AM, Vokes EE (2000) Lung cancer. Lancet 355: 479–485 10841143 10.1016/S0140-6736(00)82038-3

[bib15] Huang HF, Murphy TF, Shu P, Barton AB, Barton BE (2005) Stable expression of constitutively-activated STAT3 in benign prostatic epithelial cells changes their phenotype to that resembling malignant cells. Mol Cancer 4(1): 2 15647107 10.1186/1476-4598-4-2 PMC546221

[bib16] Kijima T, Maulik G, Ma PC, Tibaldi EV, Turner RE, Rollins B, Sattler M, Johnson BE, Salgia R (2002) Regulation of cellular proliferation, cytoskeletal function, and signal transduction through CXCR4 and c-Kit in small cell lung cancer cells. Cancer Res 62: 6304–6311 12414661

[bib17] Levine RL, Gilliland DG (2007) JAK-2 mutations and their relevance to myeloproliferative disease. Curr Opin Hematol 14: 43–47 17133099 10.1097/00062752-200701000-00009

[bib18] Levy DE, Darnell Jr JE (2002) Stats: transcriptional control and biological impact. Nat Rev Mol Cell Biol 3: 651–662 12209125 10.1038/nrm909

[bib19] Lim CP, Cao X (2006) Structure, function, and regulation of STAT proteins. Mol Biosyst 2: 536–550 17216035 10.1039/b606246f

[bib20] Maritano D, Sugrue ML, Tininini S, Dewilde S, Strobl B, Fu X, Murray-Tait V, Chiarle R, Poli V (2004) The STAT3 isoforms alpha and beta have unique and specific functions. Nat Immunol 5: 401–409 15021879 10.1038/ni1052

[bib21] Miao Z, Luker KE, Summers BC, Berahovich R, Bhojani MS, Rehemtulla A, Kleer CG, Essner JJ, Nasevicius A, Luker GD, Howard MC, Schall TJ (2007) CXCR7 (RDC1) promotes breast and lung tumor growth *in vivo* and is expressed on tumor-associated vasculature. Proc Natl Acad Sci USA 104: 15735–15740 17898181 10.1073/pnas.0610444104 PMC1994579

[bib22] Missale C, Codignola A, Sigala S, Finardi A, Paez-Pereda M, Sher E, Spano PF (1998) Nerve growth factor abrogates the tumorigenicity of human small cell lung cancer cell lines. Proc Natl Acad Sci USA 95: 5366–5371 9560282 10.1073/pnas.95.9.5366 PMC20267

[bib23] Moriguchi M, Hissong BD, Gadina M, Yamaoka K, Tiffany HL, Murphy PM, Candotti F, O'Shea JJ (2005) CXCL12 signaling is independent of Jak2 and Jak3. J Biol Chem 280: 17408–17414 15611059 10.1074/jbc.M414219200

[bib24] Muller A, Homey B, Soto H, Ge N, Catron D, Buchanan ME, McClanahan T, Murphy E, Yuan W, Wagner SN, Barrera JL, Mohar A, Verastegui E, Zlotnik A (2001) Involvement of chemokine receptors in breast cancer metastasis. Nature 410: 50–56 11242036 10.1038/35065016

[bib25] Niu G, Wright KL, Huang M, Song L, Haura E, Turkson J, Zhang S, Wang T, Sinibaldi D, Coppola D, Heller R, Ellis LM, Karras J, Bromberg J, Pardoll D, Jove R, Yu H (2002) Constitutive Stat3 activity up-regulates VEGF expression and tumor angiogenesis. Oncogene 21: 2000–2008 11960372 10.1038/sj.onc.1205260

[bib26] Opdam FJ, Kamp M, de Bruijn R, Roos E (2004) Jak kinase activity is required for lymphoma invasion and metastasis. Oncogene 23: 6647–6653 15235585 10.1038/sj.onc.1207887

[bib27] Peled A, Kollet O, Ponomaryov T, Petit I, Franitza S, Grabovsky V, Slav MM, Nagler A, Lider O, Alon R, Zipori D, Lapidot T (2000) The chemokine SDF-1 activates the integrins LFA-1, VLA-4, and VLA-5 on immature human CD34(+) cells: role in transendothelial/stromal migration and engraftment of NOD/SCID mice. Blood 95: 3289–3296 10828007

[bib28] Peled A, Petit I, Kollet O, Magid M, Ponomaryov T, Byk T, Nagler A, Ben-Hur H, Many A, Shultz L, Lider O, Alon R, Zipori D, Lapidot T (1999) Dependence of human stem cell engraftment and repopulation of NOD/SCID mice on CXCR4. Science 283: 845–848 9933168 10.1126/science.283.5403.845

[bib29] Ratajczak MZ, Zuba-Surma E, Kucia M, Reca R, Wojakowski W, Ratajczak J (2006) The pleiotropic effects of the SDF-1-CXCR4 axis in organogenesis, regeneration and tumorigenesis. Leukemia 20: 1915–1924 16900209 10.1038/sj.leu.2404357

[bib30] Reich NC, Liu L (2006) Tracking STAT nuclear traffic. Nat Rev Immunol 6: 602–612 16868551 10.1038/nri1885

[bib31] Ruffini PA, Morandi P, Cabioglu N, Altundag K, Cristofanilli M (2007) Manipulating the chemokine-chemokine receptor network to treat cancer. Cancer 109: 2392–2404 17503430 10.1002/cncr.22706

[bib32] Sanz-Rodríguez F, Hidalgo A, Teixidó J (2001) Chemokine stromal cell-derived factor-1alpha modulates VLA-4 integrin-mediated multiple myeloma cell adhesion to CS-1/fibronectin and VCAM-1. Blood 97: 346–351 11154207 10.1182/blood.v97.2.346

[bib33] Sehgal A, Keener C, Boynton AL, Warrick J, Murphy GP (1998) CXCR-4, a chemokine receptor, is overexpressed in and required for proliferation of glioblastoma tumor cells. J Surg Oncol 69: 99–104 9808513 10.1002/(sici)1096-9098(199810)69:2<99::aid-jso10>3.0.co;2-m

[bib34] Sethi T, Rintoul RC, Moore SM, MacKinnon AC, Salter D, Choo C, Chilvers ER, Dransfield I, Donnelly SC, Strieter R, Haslett C (1999) Extracellular matrix proteins protect small cell lung cancer cells against apoptosis: a mechanism for small cell lung cancer growth and drug resistance *in vivo*. Nat Med 5: 662–668 10371505 10.1038/9511

[bib35] Song L, Turkson J, Karras JG, Jove R, Haura EB (2003) Activation of Stat3 by receptor tyrosine kinases and cytokines regulates survival in human non-small cell carcinoma cells. Oncogene 22(27): 4150–4165 12833138 10.1038/sj.onc.1206479

[bib36] Soriano SF, Hernanz-Falcon P, Rodriguez-Frade JM, De Ana AM, Garzon R, Carvalho-Pinto C, Vila-Coro AJ, Zaballos A, Balomenos D, Martinez AC, Mellado M (2002) Functional inactivation of CXC chemokine receptor 4-mediated responses through SOCS3 up-regulation. J Exp Med 196: 311–321 12163560 10.1084/jem.20012041 PMC2193934

[bib37] Soriano SF, Serrano A, Hernanz-Falcon P, Martin de Ana A, Monterrubio M, Martinez C, Rodriguez-Frade JM, Mellado M (2003) Chemokines integrate JAK/STAT and G-protein pathways during chemotaxis and calcium flux responses. Eur J Immunol 33: 1328–1333 12731058 10.1002/eji.200323897

[bib38] Tamamura H, Fujisawa M, Hiramatsu K, Mizumoto M, Nakashima H, Yamamoto N, Otaka A, Fujii N (2004) Identification of a CXCR4 antagonist, a T140 analog, as an anti-rheumatoid arthritis agent. FEBS Lett 2: 99–104 10.1016/j.febslet.2004.05.056 15225616

[bib39] Vila-Coro AJ, Rodriguez-Frade JM, Martin De Ana A, Moreno-Ortiz MC, Martinez AC, Mellado M (1999) The chemokine SDF-1alpha triggers CXCR4 receptor dimerization and activates the JAK/STAT pathway. FASEB J 13: 1699–1710 10506573

[bib40] Wang J, Loberg R, Taichman RS (2006) The pivotal role of CXCL12 (SDF-1)/CXCR4 axis in bone metastasis. Cancer Metastasis Rev 25: 573–587 17165132 10.1007/s10555-006-9019-x

[bib41] Zhang XF, Wang JF, Matczak E, Proper JA, Groopman JE (2001) Janus kinase 2 is involved in stromal cell-derived factor-1alpha-induced tyrosine phosphorylation of focal adhesion proteins and migration of hematopoietic progenitor cells. Blood 97: 3342–3348 11369622 10.1182/blood.v97.11.3342

